# Complementary strategies in Psoriasis – non-pharmacological approaches for comprehensive management

**DOI:** 10.1016/j.abd.2026.501356

**Published:** 2026-05-02

**Authors:** Fernando Valenzuela, Eine Benavides, Esther Verónica Echeverry, Dan Hartmann, Daniza Bilicic

**Affiliations:** a Department of Dermatology, Universidad de los Andes, Santiago, Chile; b Department of Dermatology, Faculty of Medicine, Universidad de Chile, Santiago, Chile; c Service of Dermatology, Hospital Universitario del Valle, Cali, Colombia; d Department of Internal Medicine, Dermatology Section, Hospital Universitario del Valle “Evaristo García”, Cali, Colombia; e Faculty of Medicine, Universidad Finis Terrae, Santiago, Chile

**Keywords:** Comorbidities, Complementary therapies, Diet, Mediterranean, Exercise, Psoriasis, Vitamins

## Abstract

**Background:**

Psoriasis is a chronic immune-mediated inflammatory disease linked to systemic comorbidities such as obesity, diabetes, cardiovascular disease, and inflammatory bowel disease. Non-pharmacological interventions, such as dietary modifications, nutritional supplementation, exercise, and psychological interventions, have emerged as complementary therapies in the management of psoriasis.

**Objectives:**

Review the current and recent evidence and the role of trace elements, vitamins, diet, exercise, and psychological interventions as complementary approaches in the management of patients with psoriasis.

**Materials and methods:**

A narrative review was conducted, analyzing clinical trials, meta-analyses, and cohort studies from major databases.

**Results:**

Trace elements such as zinc, copper, and selenium, and vitamins including D, E, B-complex, and A, play roles in oxidative stress modulation, immune regulation, and keratinocyte biology. However, the clinical efficacy of micronutrient supplementation remains uncertain due to inconsistent and conflicting findings. Dietary interventions, particularly Mediterranean diet adherence and weight loss through caloric restriction or bariatric surgery, have been associated with reductions in psoriasis severity, although clear clinical protocols are lacking. Aerobic exercise appears beneficial but is underutilized, partially due to psychological and disease-related barriers. Furthermore, psoriasis is associated with a high prevalence of psychological disorders, with the necessity to integrate psychological interventions to optimize disease management.

**Study limitations:**

The available evidence is limited and with heterogeneity in study design, with small sample sizes, observational methodologies, and inconsistent intervention protocols, restricting causal inference and generalizability.

**Conclusion:**

While non-pharmacological strategies show promise as complementary interventions in psoriasis management, they cannot replace conventional therapy. Further studies are required to confirm their clinical impact. These approaches should be considered as complementary strategies, with individualized patient assessments and continuous follow-up being essential.

## Introduction

Psoriasis is a chronic inflammatory disease of immunological origin, characterized by excessive activation of the Tumor Necrosis Factor-alpha (TNF-α)/Interleukin (IL)-23/IL-17 axis, leading to hyperproliferation and abnormal differentiation of epidermal keratinocytes. This condition is frequently associated with various comorbidities, including obesity, diabetes mellitus, dyslipidemia, cardiovascular diseases, and inflammatory bowel disease.^1^ Patients with psoriasis often exhibit unbalanced dietary habits, with high-fat intake and low fiber consumption. In recent years, nutrition has been shown to play a key role in the development and progression of psoriatic disease, as well as in its associated comorbidities. This has led to a growing interest in the scientific literature regarding the use of nutritional supplements, such as trace elements and vitamins, in the treatment of psoriasis.^2^ On the other hand, exercise also influences chronic inflammatory diseases, including psoriasis. Likewise, the presence of this disease can impact patients’ physical activity levels. Regular moderate to vigorous exercise has been found to be an independent preventive factor in reducing the risk of developing psoriasis. Moreover, in overweight patients, physical activity aimed at weight loss may improve disease severity. However, this population tends to be more sedentary and faces multiple barriers to engaging in exercise.^3^ Psoriasis is not merely a dermatological condition; it significantly impacts patients' quality of life and mental health. Patients with psoriasis are 1.5 times more likely to present a mental illness, also a 12.7% can have suicidal ideations.^4^ Integrating cognitive-behavioral therapy, support groups, or other psychological interventions is also essential for a comprehensive disease management approach. In this study, the authors explore the impact of some of the most relevant nutrients in psoriasis, as well as the role of diet, exercise, and the evaluation of psychiatric conditions in its management and progression.

## Materials and methods

This study is a narrative review evaluating the role of trace elements, vitamins, diet, exercise, and psychological interventions in psoriasis pathophysiology and management. A systematic search was conducted in PubMed, Scopus, Web of Science, and Google Scholar databases, between March and April of 2025. Search terms included “Psoriasis AND trace elements (Zinc, Copper, Selenium)”; “Psoriasis AND vitamins (Vitamin D, Vitamin E, Vitamin A, Vitamin B complex)”; “Psoriasis AND diet (Mediterranean diet, Caloric restriction, Micronutrient deficiencies)”; “Psoriasis AND exercise (Physical activity, Weight loss, Obesity, Inflammation)”; “Psoriasis AND bariatric surgery”; “Psoriasis AND psychological disorders (Depression, Anxiety, Quality of life)”. Only studies published in English and Spanish were included.

## Results

### Psoriasis and trace elements

#### Zinc and copper

Zinc (Zn) is an essential cofactor in the catalytic activity of over 200 enzymes, playing a crucial role in immune function, wound healing, protein synthesis, DNA synthesis, and cell division.^5^ It acts as a coenzyme for DNA and RNA polymerases and is important in the hyperproliferation of keratinocytes observed in psoriasis skin, resulting in higher Zn consumption secondary to this accelerated cellular turnover in psoriasis, which may lead to a reduction in serum levels.^6^ Conversely, Zn deficiency can lead to decreased enzymatic activity of key antioxidants and immune cell dysfunction, thereby increasing susceptibility to viral and bacterial infections that can exacerbate skin inflammation and trigger psoriatic lesions.^7^ Copper (Cu) is another essential trace element with redox properties that make it both physiologically beneficial and potentially cytotoxic.^8^ In serum, Cu primarily binds to α2-globulin to form ceruloplasmin, a major antioxidant protein involved in scavenging excess free radicals.^7^ Elevated serum Cu levels observed in patients with psoriasis may reflect an upregulation of ceruloplasmin in response to the OS caused by the chronic inflammation in psoriasis. However, free Cu can catalyze the formation of Reactive Oxygen Species (ROS), such as superoxide anions (O_2_-), Hydrogen peroxide (H_2_O_2_), and Hydroxyl radicals (OH-) through the Fenton reaction, and contribute to cellular damage and inflammation.^9^ A 2021 case-control study involving 72 patients with psoriasis, categorized by Psoriasis Area and Severity Index (PASI) score (Group T1: PASI < 10, mild psoriasis; Group T2: PASI > 10, severe psoriasis), reported that serum Cu levels and the Cu/Zn ratio in psoriatic patients compared to healthy controls were significantly higher.^10^ The serum Zn levels were not significantly different between the two groups, suggesting that the increase in the Cu/Zn ratio may be attributable to the increase in Cu rather than decreased Zn levels. A meta-analysis conducted between 1988 and 2016 supports these findings, with studies comparing serum Cu and Zn levels between psoriasis patients and healthy controls, and they observed elevated serum Cu levels and reduced serum Zn levels in patients with psoriasis.^7^ Moreover, research in patients with psoriatic arthritis has indicated that elevated Cu and reduced Zn levels may contribute to disease pathogenesis. Interestingly, one proposed mechanism underlying the therapeutic efficacy of methotrexate in psoriatic arthritis is its ability to increase serum Zn and reduce serum Cu concentrations.^11^ In summary, although alterations in Zn and Cu levels are consistently observed in psoriatic and psoriatic arthritis, the clinical relevance of these findings and their potential utility as therapeutic targets remain unclear and require further investigation.

#### Selenium

Selenium (Se) is an essential element with antiproliferative and immunoregulatory properties. It has been proposed that Se contributes to psoriasis improvement by mitigating the OS, potentially through the upregulation of catalase and superoxide dismutase activity via its antioxidant effects.^12^ Another hypothesis suggests that Se regulates immune processes in psoriasis, modulating cytokine expression, expressing inhibitory effects on TNF-α levels and promoting an increase in CD4+ T-cells populations in the reticular dermis of psoriatic lesions.^13^, ^14^, ^15^, ^16^ Several studies have investigated the relationship between Se levels and psoriasis severity. A 2002 study reported an inverse correlation between serum Se levels and psoriasis severity.^14^ A double-blind, placebo-controlled clinical trial comparing the effects of a combination therapy of Se aspartate, coenzyme Q10, and vitamin E versus placebo demonstrated significant improvement in PASI and Severity Score (SS) in patients with severe erythrodermic and arthropathic psoriasis.^15^ Consistent with these findings, other studies have observed that serum Se levels in patients with psoriasis tend to be lower compared to healthy controls, suggesting a possible link between Se deficiency, OS, and altered immune responses in the disease’s pathogenesis.^16^, ^17^ However, evidence regarding the therapeutic efficacy of Se supplementation remains inconsistent. In a double-blind parallel-group study no added benefit of Se supplementation when its combined with narrowband UVB (NB-UVB) phototherapy compared to NB-UVB with placebo.^18^ Additionally, a case-control study conducted on hospitalized patients between January and June 2002, reported that selenomethionine supplementation was ineffective as an adjunct treatment for plaque psoriasis, and Se supplementation might contribute to sustained elevations in soluble TNF-α type 1 receptor in psoriasis patients, even after lesion remission.^19^ A 2012 meta-analysis reinforced these mixed findings, with no statistically significant differences in serum Se levels between psoriasis patients and controls.^20^ In summary, the role of Se in psoriasis appears to be multifactorial, involving potential contributions to OS regulation, cytokine modulation, and immune system balance. Despite evidence supporting an association between low Se levels and disease severity, current data on its therapeutic application remain inconclusive. Further high-quality, controlled studies are necessary to clarify the mechanistic and clinical significance of Se in psoriasis management.

### Psoriasis and vitamins

#### Vitamin D

Vitamin D (VD) plays a critical role in calcium-phosphorus homeostasis. Its prolonged deficiency leads to rickets in children and osteomalacia in adults. Beyond its skeletal effects, VD presents immunomodulatory functions, influencing both innate and adaptive immune responses.^21^ Given its role in immune regulation and skin homeostasis, the association between VD status and psoriasis has been extensively investigated.^22^, ^23^, ^24^, ^25^, ^26^ Multiple studies have reported that patients with psoriasis present lower serum concentration of 25-Hydroxyvitamin D (25(OH)D) compared to healthy controls, suggesting a potential contributory role of VD deficiency in psoriasis pathogenesis.^23^ However, interventional trials have yielded mixed results. Some randomized clinical trials evaluating oral VD supplementation have not demonstrated significant improvements in PASI scores, indicating that VD supplementation alone may not suffice to induce clinical remission.^24^ Similarly, a study examining seasonal VD supplementation during winter failed to show significant changes in disease severity between treated and placebo groups.^25^ An interventional cohort study evaluating NB-UVB therapy provides evidence that NB-UVB therapy can reduce VD-Binding Protein (DBP) and high-sensitivity C-Reactive Protein (hs-CRP) levels while increasing serum VD levels in psoriasis patients.^26^ This suggests a potential systemic anti-inflammatory effect of phototherapy, particularly in VD-deficient patients. It has also been proposed that VD derivatives may enhance phototherapy efficacy in psoriasis without causing adverse side effects.^22^, ^26^ Additionally, a Mendelian randomization analysis has suggested a potential protective effect of higher VD levels against psoriasis development, although further studies are needed to confirm this relationship.^27^ In summary, while VD appears to play a role in the pathogenesis and treatment response of psoriasis, current evidence does not support its routine use as a monotherapy. Regular monitoring of VD levels in psoriasis patients is advisable, and supplementation should be considered in individuals with confirmed deficiency.

#### Vitamin E

Vitamin E (VE) is a lipophilic antioxidant that protects cellular membranes from oxidative damage, which has been implicated in the pathogenesis of inflammatory skin diseases, including psoriasis.^28^ The relationship between VE and psoriasis has been explored in multiple studies. A meta-analysis demonstrated that serum VE levels are lower in psoriasis patients compared to healthy controls, suggesting that VE deficiency may predispose individuals to developing this immune-mediated disease.^29^ Supporting this finding, a cross-sectional study based on data from the National Health and Nutrition Examination Survey (NHANES) found that higher dietary intakes of VE were inversely associated with the risk of psoriasis.^30^ It has also been found that VE supplementation, in combination with other antioxidants such as coenzyme Q10 and Se, has improved clinical conditions in patients with severe forms of psoriasis, such as psoriatic arthritis and erythrodermic psoriasis.^31^ In conclusion, although current evidence indicates a potentially beneficial role of VE in psoriasis prevention and symptom modulation, additional high-quality clinical trials are required to establish its efficacy and determine optimal dosing strategies. At present, VE may be considered as part of a comprehensive dietary and lifestyle approach to psoriasis management, rather than as a standalone therapeutic intervention.

#### Vitamin B

Cobalamin (vitamin B12) and folic acid (vitamin B9) have been implicated in the pathophysiology of psoriasis through their role in homocysteine metabolism. Hyperhomocysteinemia in these patients has been related to VB9 and VB12 deficiency,^32^, ^33^ and with immunoinflammatory processes by activation of Th1 and Th17 lymphocytes and suppressing T-reg cells.^34^ A meta-analysis found that psoriasis patients exhibit higher homocysteine levels and a greater prevalence of hyperhomocysteinemia compared to controls; however, no significant differences in serum levels of VB12 were detected between the two groups.^32^ In contrast, another study reports a direct correlation between homocysteine levels and psoriasis severity, and an inverse relationship with folic acid levels.^35^ In a study of 98 psoriasis patients and 98 controls, which found that 57% of psoriasis patients had elevated homocysteine levels compared to 25% in controls (p < 0.0001). These patients also had significantly lower serum vitamin B12 levels, though no direct association with PASI was observed.^36^ Regarding vitamin B6, alterations in its metabolism have been suggested to influence skin inflammation, though further studies are required to confirm these findings.^37^ In summary, there is growing evidence supporting a connection between altered homocysteine metabolism and psoriasis, possibly mediated by deficiencies in VB9 and VB12. These alterations may contribute to both disease pathogenesis and its associated cardiovascular risks. While current findings highlight the potential value of assessing homocysteine and B-vitamin levels in psoriasis patients, more robust clinical trials are necessary before clear supplementation guidelines can be established.

#### Vitamin A

Vitamin A (VA) and its metabolites – such as retinoic acid and synthetic retinoid derivatives – have been used in the management of psoriasis with variable therapeutic outcomes. Retinoids influence keratinocyte proliferation, differentiation, and keratinization, processes that are dysregulated in psoriasis^38^; VA is critical for maintaining epithelial integrity and modulating immune responses. The metabolism of VA is correlated to the CYP1A1 gene. A study comparing 45 psoriasis patients and 45 healthy controls analyzed the CYP1A1 polymorphism (rs1048943) and serum VA levels. The AG genotype was found exclusively in psoriasis patients (22.2%, p = 0.001) and was associated with lower VA concentrations. Additionally, psoriasis patients had significantly reduced VA levels compared to controls (p < 0.001), suggesting that the CYP1A1 gene and VA deficiency may contribute to disease susceptibility and severity.^39^ Controversy a NHANES analysis in the United States found that psoriasis patients had higher serum VA levels compared to healthy controls, suggesting a possible association between elevated VA levels and psoriasis.^40^ However, these findings are inconsistent and require further investigation to determine the direction and implications of this association. Retinoic acid has anti-inflammatory and immunoregulatory properties, which could improve clinical psoriasis lesions. And it exhibits fungistatic effects, which are particularly relevant for psoriasis patients undergoing IL-17 inhibitor therapy, as such biologics may increase the risk of fungal infections.^41^ Topical formulations may offer a more favorable safety profile when combined with corticosteroids. Systemic retinoids, such as acitretin, have shown benefits in erythrodermic or pustular psoriasis, and have been used as an adjuvant treatment for generalized psoriasis to enhance the effects of anthralin, PUVA, or UVB therapy.^38^ In summary, although vitamin A derivatives have shown therapeutic potential in psoriasis by targeting keratinocyte function and inflammation, their clinical application is constrained by dose-dependent toxicity and variable patient response. The relationship between vitamin A levels, genetic polymorphisms, and psoriasis pathogenesis remains incompletely understood and warrants further research. Currently, VA supplementation is not recommended as a standalone treatment, but retinoids may have a role in select clinical scenarios.

### Psoriasis and exercise

Physical activity plays a crucial role in the prevention and management of chronic diseases, including inflammatory disorders, cardiovascular diseases, obesity, and metabolic syndrome.^42^ Psoriasis is associated with these comorbidities, and both obesity and physical inactivity are recognized as significant risk factors for its development. Therefore, moderate-intensity exercise has been proposed as a complementary treatment for psoriasis patients.^43^ The American Heart Association (AHA) recommend in all adults aged 18–65 years take part in moderate-intensity aerobic physical activity for a minimum of 30 minutes on 5-days each week, or vigorous-intensity aerobic physical activity a minimum of 20-minutes on 3-days each week.^44^ The HUNT study evaluated the relationship between the Body Mass Index (BMI), waist circumference, waist-to-hip ratio, and 10-year weight changes on psoriasis risk. The study found a significant association between an increase in body weight and psoriasis risk, particularly in individuals who gained 10 kg or more during the follow-up period. These findings highlight weight control as a potential preventive strategy for psoriasis.^45^ It has been demonstrated that increased adipose tissue affects levels of inflammatory cytokines involved in psoriasis, such as TNF-α and IL-17. Since obesity is a risk factor for developing or worsening psoriasis, physical activity may have a protective role against the disease.^46^ A randomized clinical trial by Naldi et al. evaluated the impact of exercise in psoriasis patients with PASI > 10 and overweight or obesity. The study included 303 patients who performed aerobic exercise for at least 40-minutes, three times per week, for 20-weeks, aiming for a 5% weight reduction. The exercise group experienced a 48% PASI reduction, while the control group showed a 25.5% PASI reduction (p = 0.02).^47^ A recent systematic review examined the role of physical activity in preventing and treating patients with psoriasis. The findings suggest that engaging in moderate intensity exercise can lead to improved antioxidant gene expression, reduced oxidative stress, higher levels of sex hormone-binding globulin, and lower levels of Insulin-like Growth Factor 1 (IGF-1), along with a decrease in adipose tissue mass. These changes in metabolism and hormones help lower insulin and leptin levels, boost adiponectin levels, and ultimately reduce systemic inflammation.^48^ A 2022 systematic review suggested that the beneficial effects of exercise on psoriasis may be mediated, in part, through adipose tissue reduction. However, psoriasis patients often report reduced physical activity levels, attributed to disease-related limitations such as skin discomfort, pain, or social stigma.^45^ In another study that evaluated the barriers to physical activity in patients with chronic psoriasis, found that 53% of patients aged 18–65, and 66% of those over 65, did not meet recommended physical activity levels for cardiovascular health, the main key barriers were skin sensitivity and discomfort during exercise, embarrassment about the appearance of their skin, limitations in clothing choices (such as avoiding sportswear that exposes affected areas), and the impact of treatments interfering with exercise routines. The study also showed that greater disease severity and poorer dermatology-related quality of life (measured by the DLQI) were linked to lower physical activity levels, particularly in women aged 18–65.^49^ In summary, physical activity may play a dual role in both psoriasis prevention and symptom improvement, particularly among overweight or obese individuals. It should be considered as a complementary, non-pharmacological strategy within a multidisciplinary treatment approach. Also, healthcare professionals need to recognize and address the specific barriers that can restrict psoriasis patients from engaging in physical activity. Tailored interventions that accommodate the unique challenges faced by individuals with psoriasis are recommended to promote healthier, more active lifestyles.

### Psoriasis and diet

The role of diet as a treatment for psoriasis has been evaluated in several studies, showing that a dietary intervention can reduce systemic inflammation through the intake of antioxidant and anti-inflammatory nutrients.^50^ The current Western diet is considered pro-inflammatory, being rich in omega-6 fatty acids, high-calorie intake, and trans fats, and it may exacerbate immune dysregulation in psoriasis.^51^ In contrast, nutritional strategies that promote immune homeostasis, particularly the Mediterranean diet, rich in vegetables, fruits, whole grains, and healthy fats, have been associated with reduced incidence of metabolic and inflammatory disease and may offer a protective effect in psoriasis.^52^ Calorie-restricted diets have been shown to slightly reduce PASI scores and improve the quality of life in affected individuals.^53^ Furthermore, diets high in fiber, vitamins, and polyphenols have demonstrated anti-inflammatory properties and may positively influence the gut microbiota, a factor increasingly recognized in psoriasis pathophysiology.^53^, ^54^, ^55^ A comparative study of 45 psoriasis patients with 43 controls, revealing that psoriasis patients had higher BMI, LDL cholesterol, and total cholesterol levels, but lower HDL cholesterol levels. Additionally, they consumed more carbohydrates and fats, but less fiber, folate, and VE. These findings suggest that nutritional imbalances and dyslipidemia may contribute to disease severity, reinforcing the importance of targeted dietary counseling in psoriasis management.^56^ A meta-analysis found that weight loss through lifestyle interventions significantly improved psoriasis compared to control group interventions. Consequently, it has been suggested that, in combination with conventional therapy, an appropriate diet should be implemented to enhance clinical responses in psoriasis and reduce comorbidities.^57^

### Psoriasis and bariatric surgery

Bariatric surgeries, including procedures such as sleeve gastrectomy and gastric bypass, have emerged as a potential complementary therapy in obese psoriasis patients. A recent observational study of 32 patients undergoing bariatric surgery reported a significant reduction in PASI score after the surgical intervention.^58^ In another study involving 10 obese psoriasis patients, 70% remained in remission for at least six months following surgery, and three out of four patients on systemic therapy discontinued medication due to significant improvement. Improvements in quality of life and reduction in cardiovascular risk factors were also observed.^59^ However, bariatric surgery is associated with an increased risk of micronutrient deficiencies, including VD, VB12, iron, calcium, Se, Zn, among others, due to altered nutrient absorption post-surgery.^60^, ^61^, ^62^, ^63^ These deficiencies can lead to anemia, osteoporosis, neurological complications, and potentially exacerbate psoriasis symptoms. For example, selenium deficiency has been linked to muscle weakness, cardiomyopathy, and psoriasis flares.^63^, ^64^ Despite the widespread use of multivitamin and mineral supplements, nutrient deficiencies persist in a considerable percentage of patients, highlighting the need for continuous evaluation.^63^, ^65^ In conclusion, weight reduction strategies, including diet and bariatric surgery, may offer substantial benefits in psoriasis ‒ particularly among obese patients. Nevertheless, these interventions require careful patient selection and a comprehensive risk-benefit evaluation, given the potential for long-term metabolic and nutritional complications.

### Psoriasis and mental health

It’s clear that psoriasis is associated impacts in patients' quality of life and mental health. The presence of depression, anxiety, and social stigma is well-documented in psoriasis patients. In a systematic review of anxiety disorders in patients with psoriasis was 7%–48%.^66^ In the case of depression, the prevalence is estimated to 20%–30%.^67^ The association with other psychiatric conditions has been described, such as schizophrenia, bipolar disorder, and posttraumatic stress disorder.^4^ The suicidal ideation in patients with psoriasis has been reported in a 12.7% of the patients.^68^ The higher presence of proinflammatory markers has been correlated with elevated risk of depression, anxiety, and schizophrenia in patients with psoriasis, suggesting a nexus with the neuroinflammatory pathways and the severity of lesions in the skin.^69^ In the other hand, social stigma affects patients with psoriasis, potentially leading to worsened symptoms or difficulty engaging in physical activity.^70^, ^71^ In patients with psoriasis, a screening for depression, anxiety, and suicidality should be made, and the evaluation of other psychiatric conditions. Addressing stress, depression, or anxiety can improve the overall health and quality of life of patients, reducing the severity of skin lesion, the consideration of behavioral interventions or Cognitive-Behavioral Therapy (CBT) might aid in improving adherence to dietary changes and exercise programs.

## Discussion

Psoriasis is an immune-mediated inflammatory chronic disease caused by a complex interplay of genetic, environmental, and lifestyle factors.^1^ Emerging evidence highlights the role of nutrition, physical activity, and specific micronutrients in modulating the disease course and associated comorbidities.^31^

For instance, Se shows potential due to its antioxidant and immunoregulatory functions; studies report inconsistent outcomes, with some suggesting clinical improvements and others finding no significant effect.^20^ VD deficiency is frequent in psoriasis patients, yet supplementation alone has not consistently translated into clinical improvement, possibly due to differences in baseline levels, dosing, or concurrent therapies.^27^ Similar ambiguities surround VE, with a lack of definitive evidence for its use as a monotherapy. The relationship between elevated homocysteine and psoriasis implicates deficiencies in vitamin B12 and folic acid. Although some studies demonstrate correlations with disease severity and cardiovascular risk, direct benefits from supplementation remain uncertain.^32^, ^33^ Vitamin A and its derivatives have shown efficacy in certain psoriasis subtypes but are limited by their toxicity profiles. Genetic polymorphisms influencing VA metabolism may further affect individual responses.^39^ Exercise and dietary interventions offer promising adjunctive benefits. Structured aerobic exercise has demonstrated improvements in PASI scores and metabolic parameters,^47^ yet patient adherence may be hindered by disease-related physical or psychological barriers.^49^ Similarly, while anti-inflammatory diets ‒ particularly the Mediterranean diet ‒ are associated with symptom improvement, adherence and long-term outcomes require further validation.^52^ Caloric restriction and nutrient-rich diets improve quality of life and inflammation, but need standardization in clinical recommendations. Bariatric surgery provides significant benefits for obese psoriasis patients, including symptom remission and medication reduction. However, the procedure is associated with long-term micronutrient deficiencies (e.g., VD, Se, B12), necessitating careful post-operative monitoring.^62^, ^63^ The limited sample sizes and observational nature of current studies also limit the strength of conclusions. Psoriasis significantly impacts mental health, with a high prevalence of anxiety, depression, and even suicidal ideation.^4^ It is also associated with other psychiatric disorders and inflammatory processes.^69^ Additionally, social stigma can worsen symptoms. Therefore, psychological support and interventions like cognitive-behavioral therapy are essential.

Despite promising associations, several limitations constrain the current body of research. Firstly, many studies cited are observational or cross-sectional, which limits causal inference. Randomized controlled trials remain scarce or yield mixed results, particularly for micronutrient supplementation. Variability in study design, sample sizes, disease severity, and intervention protocols further complicates data interpretation. Additionally, heterogeneity in patient populations, such as age, BMI, comorbidities, and baseline nutrient levels, affects generalizability. Confounding factors such as medication use, dietary patterns, and lifestyle behaviors are often inadequately controlled. Future research should prioritize randomized controlled trials, standardized protocols for dietary, exercise, and psychological interventions, and integration of psychosocial support into psoriasis management strategies.

In summary, while the role of nutrition and lifestyle in psoriasis management is gaining recognition, the current evidence is insufficient to establish universal clinical guidelines. Individualized strategies focusing on weight management, balanced nutrition, and regular moderate-intensity physical activity hold promise in enhancing treatment outcomes and quality of life for patients with psoriasis.

## Conclusion

There is evidence suggesting an interaction between trace elements, vitamins, diet, exercise, and psychological support with psoriasis. This information offers promising complementary strategies for psoriasis management. However, given the variability and inconsistency of the current evidence, these approaches should not substitute for established treatments. Further studies and research are needed to clarify these relationships and aim to develop clear clinical guidelines and integrate psychological considerations to optimize outcomes in psoriasis patients.

## ORCID ID

Fernando Valenzuela: 0000-0003-1032-9347

Eine Benavides: 0000-0002-2064-2647

Esther Verónica Echeverry: 0000-0002-5344-1251

Daniza Bilicic: 0000-0002-1421-8989

## Research data availability

Does not apply.

## Financial support

None declared.

## Authors' contributions

Fernando Valenzuela: Approval of the final version of the manuscript; critical literature review; manuscript critical review; preparation and writing of the manuscript.

Eine Benavides: Critical literature review; manuscript critical review; preparation and writing of the manuscript.

Esther Verónica Echeverry: Critical literature review; manuscript critical review; preparation and writing of the manuscript.

Dan Hartmann: Approval of the final version of the manuscript; critical literature review; preparation and writing of the manuscript.

Daniza Bilicic: Critical literature review; preparation and writing of the manuscript.

## Conflicts of interest

None declared.
